# Optimization of DMD-based independent amplitude and phase modulation by analysis of target complex wavefront

**DOI:** 10.1038/s41598-022-11443-x

**Published:** 2022-05-11

**Authors:** Alexandra Georgieva, Andrey V. Belashov, Nikolay V. Petrov

**Affiliations:** 1 Digital and Display Holography Laboratory, ITMO University, Kronverksky 49, St. Petersburg, 197101 Russia; 2 Ioffe Institute, 26 Politekhnicheskaya, St. Petersburg, 194021 Russia

**Keywords:** Imaging and sensing, Adaptive optics

## Abstract

The paper presents the results of a comprehensive study on the optimization of independent amplitude and phase wavefront manipulation which is implemented using a binary digital micromirror device. The study aims to investigate the spatial resolution and quantization achievable using this approach and its optimization based on the parameters of the target complex wave and the modulation error estimation. Based on a statistical analysis of the data, an algorithm for selecting parameters (carrier frequency of binary pattern and aperture for the first diffraction order filtering) that ensures the optimal quality of the modulated wavefront was developed. The algorithm takes into account the type of modulation, that is, amplitude, phase, or amplitude-phase, the size of the encoded distribution, and its requirements for spatial resolution and quantization. The results of the study will greatly contribute to the improvement of modulated wavefront quality in various applications with different requirements for spatial resolution and quantization.

## Introduction

The synthesis of wavefronts with known characteristics has attracted the interest of many researchers in the field of photonics. Some of the applications of wavefront shaping are high-resolution microscopy^1^, laser beam shaping^2,3^, scattering media characterization^4–6^, holographic displays^7^, quantum cryptography^8^, metrology^9^, compressed sensing^10^, 3D bioprinting and lithography^11^. To date, there exists a range of static and dynamic wavefront modulators, such as diffraction optical elements^12^, metasurfaces^13^, adaptive optical elements^14^, which provide the possibility to operate with the amplitude, phase, or polarization of the beam profile in a wide range of wavelengths^15,16^. Adaptive spatial light modulators with programmable precise control of the wavefront have become a valuable tool for various applications, e.g., in imaging systems^17^. Two major types of such devices can be outlined: liquid crystal-based spatial light modulators and micro-electromechanical systems (MEMS). The former includes such subtypes as transmissive liquid crystal, reflective liquid crystal on silicon, and ferroelectric liquid crystal. MEMS-based spatial light modulators are presented by a digital micromirror device (DMD), an active micromirror matrix, and a grating light valve^18^.

Each of the devices is characterized by the type of modulation, among which they can be distinguished: amplitude-only, phase-only, and simultaneous amplitude-phase modulation. Different types of modulators were compared, based on which the advantages and disadvantages of each technique were identified^18–21^. The choice of the required device is determined by the peculiarities of the problem to be solved in a particular case. Several important characteristics of wavefront modulators can be highlighted: the speed of operation, modulation dynamic range, number and size of pixels, and modulation efficiency. In applications where high speed is required and spatial resolution can be sacrificed to achieve high light modulation rates, the use of DMD is preferable due to its high refresh rate^22^. In addition, DMD constructively assumes only binary modulation. Compared to other modulators, DMD has a high switching speed, a high fill-factor (90%), and a relatively low cost^23–25^. Over the past few years, such devices have been actively used in various studies^11,22^, and commercial devices (e.g., holotomographic microscope HT-1H, developed by Tomocube, Inc). It provides a high enhancement factor in the task of focusing through the scattering medium^19^ or improving contrast and beam-shaping fidelity in optical imaging^20^. This is particularly relevant in biomedical applications where rapid processes are involved, or the possibility of real-time measurement should be provided^11,22,26^. DMD consists of a CMOS-placed micromirrors array, each of which can have only two stable states: “On” ($+12^{\circ }$) and “Off” ($-12^{\circ }$)^22^. Each micromirror represents a single pixel of the projected image. In addition, the use of binary (1-bit) holograms is convenient in terms of data capacity, for example, for implementation in holographic displays^27^. Another advantage of binary holograms over grayscale holograms is also that they can be easily printed^28^.

Various approaches have previously been proposed for generating binary DMD-patterns or converting grayscale holograms into binary holograms in order to ensure amplitude-phase modulation, for instance, global and local thresholding methods^29^, iterative techniques^30,31^, error diffusion method^32^, the superpixel-based method^33^, and off-axis computer-generated Lee holography technique^34^. The latter is an efficient and fast method, especially eligible for ultrafast radiation modulation^35^. In this method, the first diffraction order Fourier filtering with an aperture is utilized, which affects spatial resolution. The number of available amplitude levels (or image quantization) depends on the DMD-generated pattern parameters^36^. These factors significantly affect the image quality. Various methods of quantization and resolution improvement were proposed. The study by Reimers et al.^37^ elucidated the optimal resolution obtained for the detection of objects with different requirements for spatial resolution in terms of hyperspectral imaging. The results reported by Zhang et al.^38^ suggest that in single-pixel imaging, the quantization error caused by binarization can be eliminated by error diffusion dithering and a high ratio of upsampling. In their recent study, Chipala and Kozacki^7^ demonstrated the correlation between DMD dispersion and holographic image resolution and proposed a method to improve image quality. However, the area of achieving maximum quantization and resolution values by optimizing the experimental setup and binarization parameters has not been explored in depth. Moreover, the influence of the type of modulation (amplitude, phase, or amplitude-phase), differences in the number of points in the encoded distribution, and cutoff frequency DMD have not been analyzed in extensive studies. Meanwhile, in this study we demonstrate that different binary patterns are required for optimal wavefront modulation in dependence on the target complex wave parameters and particular criteria imposed on the modulated wavefront. In certain types of applications either spatial resolution or quantization of the target complex wave may be the most important characteristic. For instance, some imaging applications of wavefront modulation, such as successful data transfer, targeted fluorescence proteins activation/desactivation, optogenetic stimulation, structured illumination fluorescence microscopy^39–42^, require the generation of amplitude or/and phase distributions with high spatial resolution. On the other hand, when the application of the wavefront modulation system is aberration correction^43^ or generation of a uniform amplitude distribution, the quantization of both phase and amplitude distribution should be taken into account to provide high-performance wavefront modulation with high dynamic range and quantization^44,45^. In some applications such as the implementation of ptychographic approaches^46^, both parameters may be important, and a certain trade-off between these characteristics should be found in each particular case.

Here, we develop and implement a numerical model for the simulation of DMD-based complex wave modulation and the evaluation of its accuracy. A statistical study was performed, which reveals the dependence between the wavefront modulation quality and such parameters of the target wave as its size, modulation type (phase/amplitude/amplitude-phase), and the requirements of spatial resolution and quantization. The algorithm was developed for the determination of the optimal modulation parameters, i.e., the carrier frequency of the binary pattern and the aperture for the first-order diffraction filtering. In addition, experimental validation of the proposed optimization method was performed.

In the subsequent sections, we describe an experimental setup and the basic principles of independent amplitude and phase wavefront modulation using DMD. This is followed by a discussion of the criteria for modulated wavefront quality estimation. The results section demonstrates the statistical study on the modulation type and target distribution size for the optical system optimization for independent amplitude and phase wavefront manipulation. Next, we performed the experimental evaluation of the developed approach using a random and optimized DMD pattern. In conclusion, we briefly summarize our results and provide practical applications for the developed optimization algorithm.

## Methods

### Experimental setup

**Figure 1:**
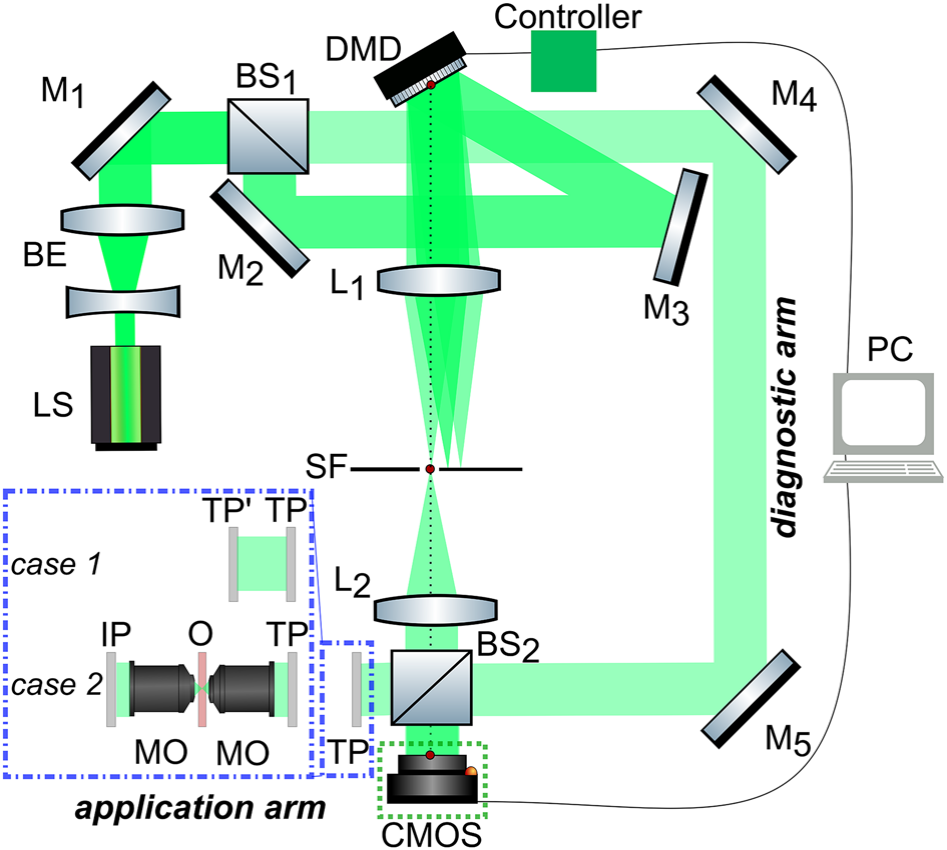
Experimental setup scheme. BE is a beam expander, M$_{1-5}$ are mirrors, S is a shutter, BS$_{1-2}$ are beam splitters, L$_{1-2}$ are lenses, SF is a spatial filter, TP is a target plane, CMOS is matrix detector, MO are objectives, IP is an image plane, O is an object.

The experimental setup is presented by the Mach-Zender interferometer with DMD and 4-f lens system in the object beam (Fig. 1).

This setup provides an opportunity to modulate the wavefront using DMD, as well as to evaluate its modulation quality by image reconstruction with off-axis digital holograms. DMD was operated using controller DLPC900 (modulation frequency is 9 kHz). The laser beam with the wavelength of 532 nm was increased in diameter by the beam expander BE, then it was divided into object and reference waves by the beam splitter BS$_1$. The object beam was incident on the DMD (DLP6500FYE Texas Instrument Light Crafter with 1920 $\times$ 1080 micromirrors with a size of $7.56$ μm), where the corresponding pattern was displayed on the matrix. Then it was focused with the lens L$_1$ on the Fourier plane, where the first diffraction order was separated by the spatial filter SF with a slit-type aperture size scaleable along the *x* coordinate. Afterward, the lens L$_2$ collimated the radiation. The lenses L$_1$ and L$_2$ formed the 4f-system. The desired field distribution was formed in the target plane TP after the second beamsplitter BS$_2$. This plane is the output image plane of the 4f-system. The presence of BS$_2$ ensured the location of this plane simultaneously both at the end of the diagnostic arm, namely, in the sensor plane (CMOS) and in the application arm.

The beam splitter BS$_2$ enabled the implementation of simultaneous monitoring of the modulated wave using CMOS sensor in the monitoring arm (indicated with green dotted frame in Fig. 1) and the modulated wave utilization for any research purposes in the application arm (indicated with blue dash-dotted frame in Fig. 1). When the shutter S is opened, the plane reference wave passes through the mirrors M$_{4}$ and M$_{5}$ and enables the detection of the formed amplitude and phase distributions in the application arm. Holographic monitoring was implemented in the following way: wave modulated with DMD and a plane reference wave were incident on the sensor of CMOS camera at a small angle, thus forming an off-axis digital hologram. Then we used the local least square estimation algorithm^47–49^ for complex wave reconstruction.

It should be noted that some applications may require the target wavefront of a specific structure to be obtained beyond the target plane (TP). Such a complex wave located in an arbitrary plane may be engineered precisely by means of an analysis of the resulting field at TP in the diagnostic arm and solving the diffractive equations which describe the wavefront propagation beyond the TP in backward direction^50^ (case 1 in Fig. 1). Then, this information should be included in the feedback loop: the calculated wavefront distribution is set as the target one, taking into account possible error correction procedures to minimize the mismatch between the target and actually generated wavefront in TP. After the wavefront synthesis in TP, its further physical diffraction propagation forms the initially desired wavefront at a certain plane. The numerical displacement of the target wavefront formation plane was numerically and experimentally investigated in^51^. The alternative is not to propagate wavefront numerically, but to image TP further by an additional telecentric system with custom magnification^52^ (case 2 in Fig. 1). It is also necessary to mention that independent values in the amplitude and phase distributions can only be obtained in TP or in the corresponding image plane which is a result of the 4-*f* system projection. This fact results from the phase distribution of the coherent wave affecting the amplitude distribution and vice versa due to diffraction.

### Principles of binary complex wavefront modulation

In this subsection, the basic principles of independent amplitude and phase modulation of the wavefront using DMD generated binary off-axis digital hologram are considered and discussed. Lee proposed one of the possible approaches for independent modulation of amplitude and phase^34^. The method consists in generating a binary pattern in the form of a synthetic off-axis hologram and its further reconstruction.

The fringes in an analog hologram can be used to change the phase and amplitude of the incident light wave, thus producing an image of the recorded object. Variation of binary fringe parameters such as width and periodicity in the formed binary DMD pattern enables the manipulation of amplitude and phase distributions. The approach to perform the independent amplitude-phase modulation from binary amplitude modulation is based on the spatial filtering of the wave in one of the diffraction orders arising from the reflection of an incident wave from DMD. It can be conducted, for example, by spatial filtration of this diffraction order if the binary pattern is an off-axis hologram with high enough carrier frequency.

Experimentally it is usually implemented by Fourier transform performed using a concave lens (L$_2$), as demonstrated in Fig. 1. Filtration of the first diffraction order using adjustable aperture (SF) with inverse Fourier transform by another concave lens L$_2$ enables the reconstruction of the target wave modulated by DMD.

The spatial frequency of the binary hologram is calculated using the carrier frequency and phase change as follows^34^:

1 $$\begin{aligned} \begin{aligned} \nu _x(x,y)=k_x+\frac{1}{2\pi }\frac{\partial \phi (x,y)}{\partial x}\\ \nu _y(x,y)=k_y+\frac{1}{2\pi }\frac{\partial \phi (x,y)}{\partial y} \end{aligned} \end{aligned}$$

where $k_x$ and $k_x$ are carrier frequencies in the *x* and *y* directions, which are inversely proportional to the specified fringe period due to the wavefront tilt. The second summand refers to the spatial frequency of the wave with target phase distribution.

**Figure 2:**
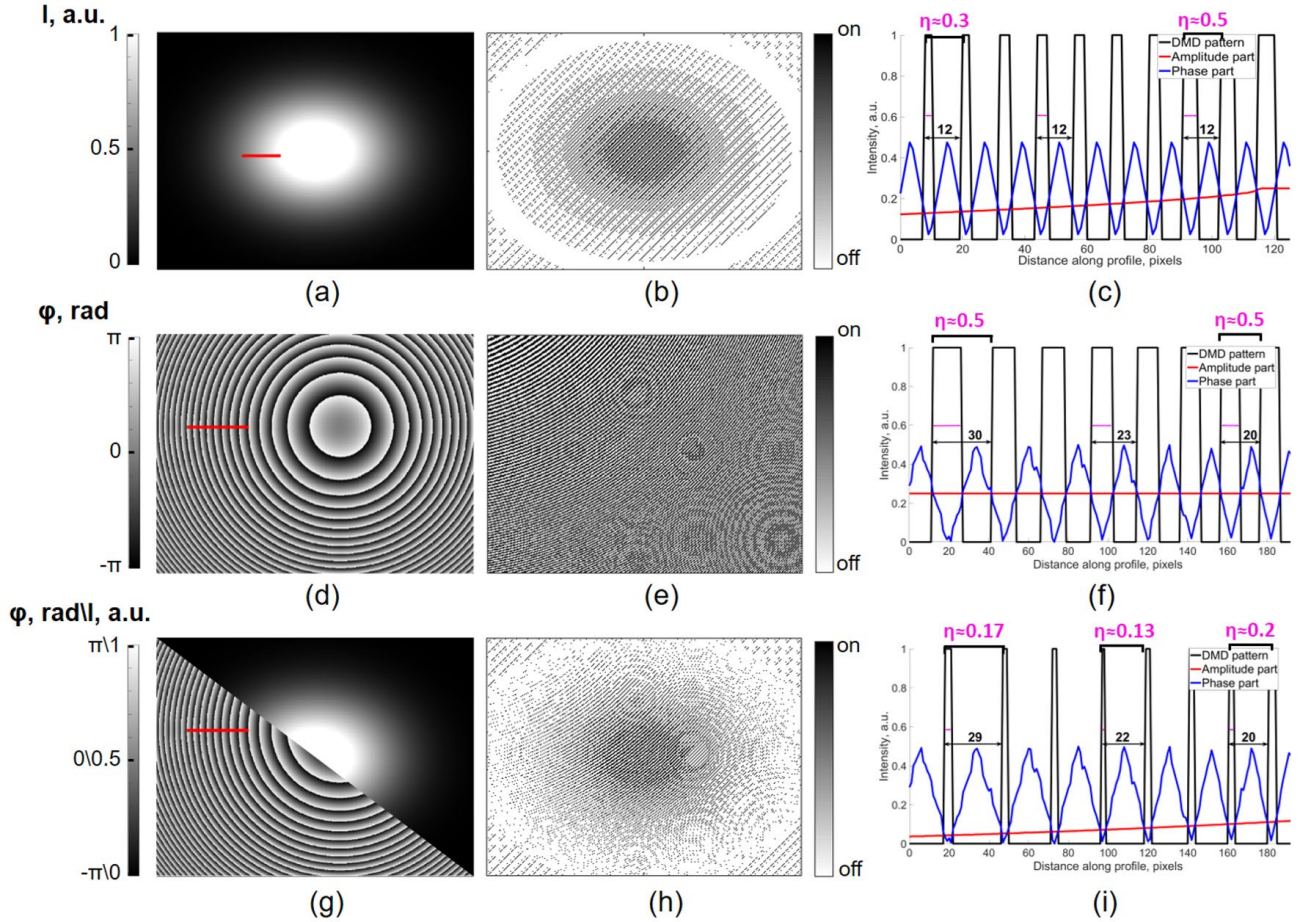
Demonstration of the independent modulation of the amplitude (**a**–**c**), phase (**d**–**f**), and amplitude-phase (**g**–**i**). The target amplitude (**a**), phase (**d**), and amplitude-phase (**g**) distributions are presented in the first column. The red line indicates the coordinates of the plot in (**c**,**f**,**i**); The resultant DMD patterns with carrier frequency $\frac{2\pi }{12}$ (**b**,**e**,**h**) are shown in the second column; (**c**,**f**,**i**) the plot of DMD pattern function (black line), phase part (blue line) and amplitude part (red line) are shown in the third column.

A DMD-pattern *h*(*x*, *y*) was formed using the limiter to obtain the binarization of amplitude hologram^34^:

2 $$\begin{aligned} h(x,y)= {\left\{ \begin{array}{ll} 1; \quad \left| \left( \left[ \frac{\phi (x,y)}{2\pi }+(k_x \cdot x + k_y \cdot y)\right] \mod 1\right) -0.5\right| \le \frac{asin|A(x,y)|}{2\pi } \\ 0; \quad \text {otherwise} \end{array}\right. } \end{aligned}$$

where *A*(*x*, *y*) is the target amplitude, $\phi (x,y)$ is the target phase, $k_x$ and $k_y$ are the carrier frequencies in DMD pixels of the binary off-axis hologram in the *x* and *y* direction. The left part of the inequality (2) is responsible for the encoding of the phase distribution, while the amplitude distribution is encoded by the right part. To show the segregation of amplitude and phase parts, we consider DMD-patterns simulated for amplitude-type, phase-type, and amplitude-phase modulations (Fig. 2). By the type of modulation in this case we mean an amplitude-only, phase-only, or simultaneous independent amplitude and phase wavefront variation in the TP plane.

The first example of varying amplitude (2D Gaussian distribution) and constant phase distributions is presented in Fig. 2a–c. The target amplitude image (Fig. 2a and the simulated binary pattern (Fig. 2b) demonstrate how the complex wave amplitude can be modulated by the variation of the relative amount of DMD pixels in On-state (the On-pixels). The right (amplitude) and left (phase) parts of the inequality are represented by red and blue lines, respectively. The left part of the inequality representing the phase distribution is a simple periodical function due to the linear increase of $\frac{\phi (x,y)}{2\pi }+(k_x \cdot x + k_y \cdot y)$ term and “*mod*1” operation due to the phase distribution consistency. However, the target amplitude image is changed along *x* coordinate (red line), thus varying the relative amount of the On and Off pixels. If the amplitude part is larger than the phase part, the pixel is On, otherwise it remains Off. In this case, the ratio between the On pixels and the total number of pixels per one period (the On pixel occupancy coefficient $\eta$) is changed from 0.3 to 0.5, while the target amplitude increases approximately from 0.13 to 0.25. Due to the constancy of the phase distribution, the carrier frequency and the spatial frequency value of the binary hologram are constant and equal $\frac{2\pi }{12}$. Subsequently, the fringe period is 12 DMD pixels (indicated with black arrows in Fig. 2c).

The phase-only variation is demonstrated with the target spherical phase distribution and constant amplitude. Fig. 2d–f show the target phase distribution (Fig. 2d), the simulated DMD pattern (Fig. 2e) and the plotted functions of DMD pattern pixels, amplitude part and phase part (Fig. 2f) of inequality (2) in the case of phase-only modulation. In this example, the right part of the inequality (2) is constant due to the absence of the modulation of amplitude. However, variation of the target phase along *x* axis results in the change of $\frac{\phi (x,y)}{2\pi }+(k_x \cdot x + k_y \cdot y)$ slope and alteration of the phase part periodicity and spatial frequency of binary hologram. The black arrows in Fig. 2f demonstrate how the phase variation results in the change of the fringe period from 29 to 20 pixels. At the same time, $\eta$ is constant among the binary hologram and equal to 0.5.

Therefore, phase modulation is achieved by the variation of the binary fringe period, whereas the amplitude modulation is obtained due to the variation of the On-pixel occupancy coefficient. As it is possible to separately vary these two parameters in each local area of the image, the independent manipulation of the phase and amplitude distributions can be achieved.

An example of independent modulation of both amplitude and phase parts of the complex wavefront is presented in Fig. 2g–i. It can be noted that both fringe periodicity and the On pixel occupancy coefficient are changed due to amplitude and phase variation.

### Modulation error estimation

Quantitative assessment of the modulated wavefront quality can be achieved by calculating the root mean square error (RMSE) between the true target distribution and the resulting modulated wavefront. To perform optimization and find the optimal parameters, we numerically simulated DMD-based target wavefront modulation with various carrier frequencies and filtration aperture sizes and calculated the RMSE of the target amplitude or phase distributions using the following equation:

3 $$\begin{aligned} RMSE = \sqrt{\frac{1}{n}\sum _{i=1}^n{(x_t-x_r)}^2} \end{aligned}$$

where *n* is the number of pixels of distribution, $x_t$ is the value of the target amplitude or phase in some pixel, $x_r$ is the value of the resulting amplitude or phase in some pixel. Minimization of the calculated error allows us to obtain the optimal parameters of the DMD-generated binary pattern for a particular target complex wave.

**Figure 3:**
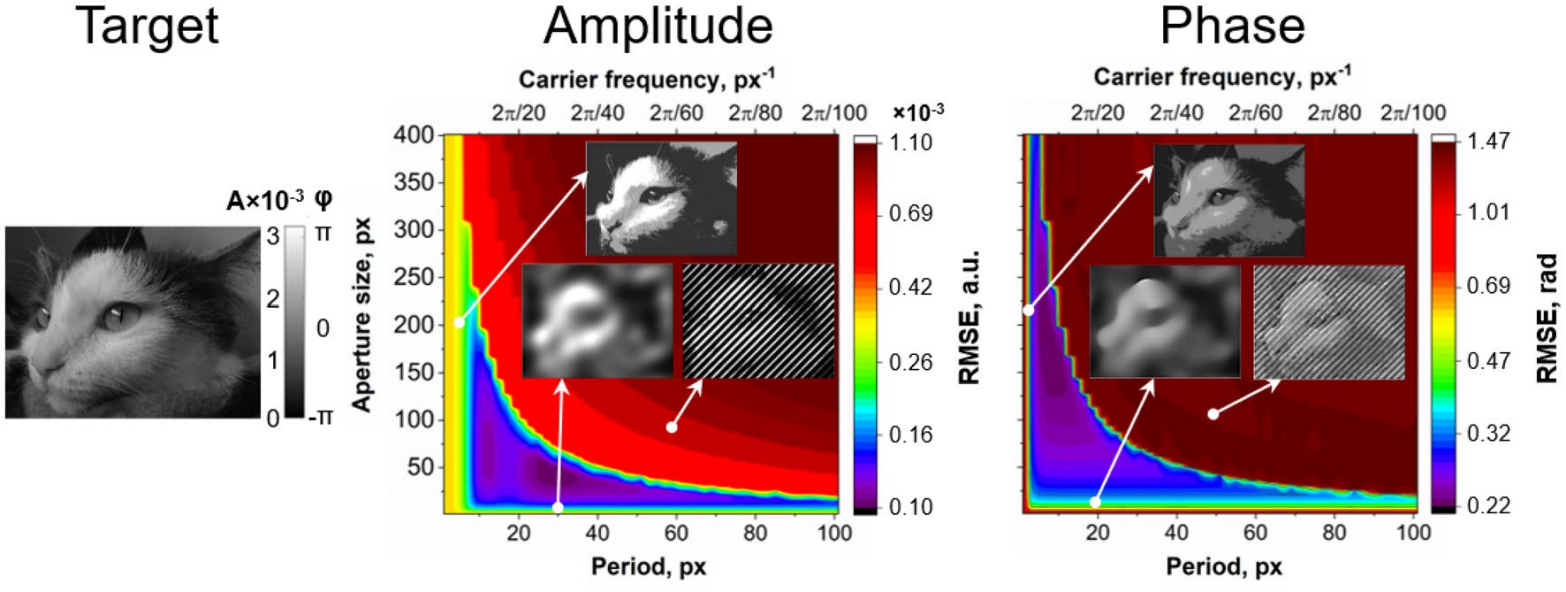
Amplitude and phase modulation RMSE maps dependent on aperture size and fringe period for the “cat” object. Insets: obtained intensity and phase distributions at the points indicated by the markers. Target distribution is shown on the left.

To investigate the dependence of the modulation quality on the major experimental scheme parameters, each of these images was encoded with the aperture size ranging from 1 to 400 pixels and the fringe period varying from 1 to 100 pixels. Note that in our numerical simulation the size of the spatial filtration aperture is in relative values (pixels within the Fourier domain). Physical value of the spatial aperture in experimental work should be recalculated taking into account focusing distance of $L_1$ (see Fig. 1) and DMD physical size. The location of every i-th pixel of DMD in the focal plane of $L_1$ can be calculated as $i\frac{f_1 \lambda }{N\Delta _p}$, where $f_1$ is focal distance of $L_1$, $\lambda$ is wavelength, *N* is linear matrix size and $\Delta _p$ is pixel size. For each of the obtained images, its RMSE from the target wave was calculated and represented as a 2D pseudocolored surface (see Fig. 3). Analysis of such a 2D wavefront modulation error map makes it possible to determine the optimal parameters of the complex wave modulation.

The resulting amplitude and phase wavefront modulation error maps are separated on high and low error areas with a hyperbola-type curve, above which all resulting distributions have critical defects in the form of interference fringes, caused by the overlay of two (or more) adjacent diffraction orders into the aperture. Utilizing small apertures also leads to defects of the modulated wavefront due to excessive spatial filtering, resulting in the decrease of spatial resolution and image blurring. The area of high apertures and low fringe periods also leads to defects expressed in the form of sharp shade transitions because of the limited number of gradations. The detailed discussion of the optical system parameters influence on the wavefront quality is presented in Suppl. 1. Therefore, to determine the optimal modulation parameters, it is necessary to consider several factors, which will be discussed below.

In case of simultaneous amplitude and phase modulation the modulation error can be calculated as follows: $\delta =1-F$, where $F=|E^*_{target} E_{obtained}|^2$, $E_{target}$ is a target field, $E_{obtained}$ is the field obtained after DMD modulation^33^.

## Results

### Target diversity study

This section discusses the main factors influencing the choice of optimal parameters, i.e. aperture size and fringes period or carrier frequency of binary hologram, to ensure the best quality of the modulated complex wave distributions. Note that due to the complexity of target amplitude and phase distributions, it is difficult to quantify the spatial resolution and quantization level requirements in the general case. Therefore, accurate estimation of high and low spatial frequencies contribution can be performed using a numerical model only, although the analytical approach can be applied as well in a limited number of cases.

**Figure 4:**
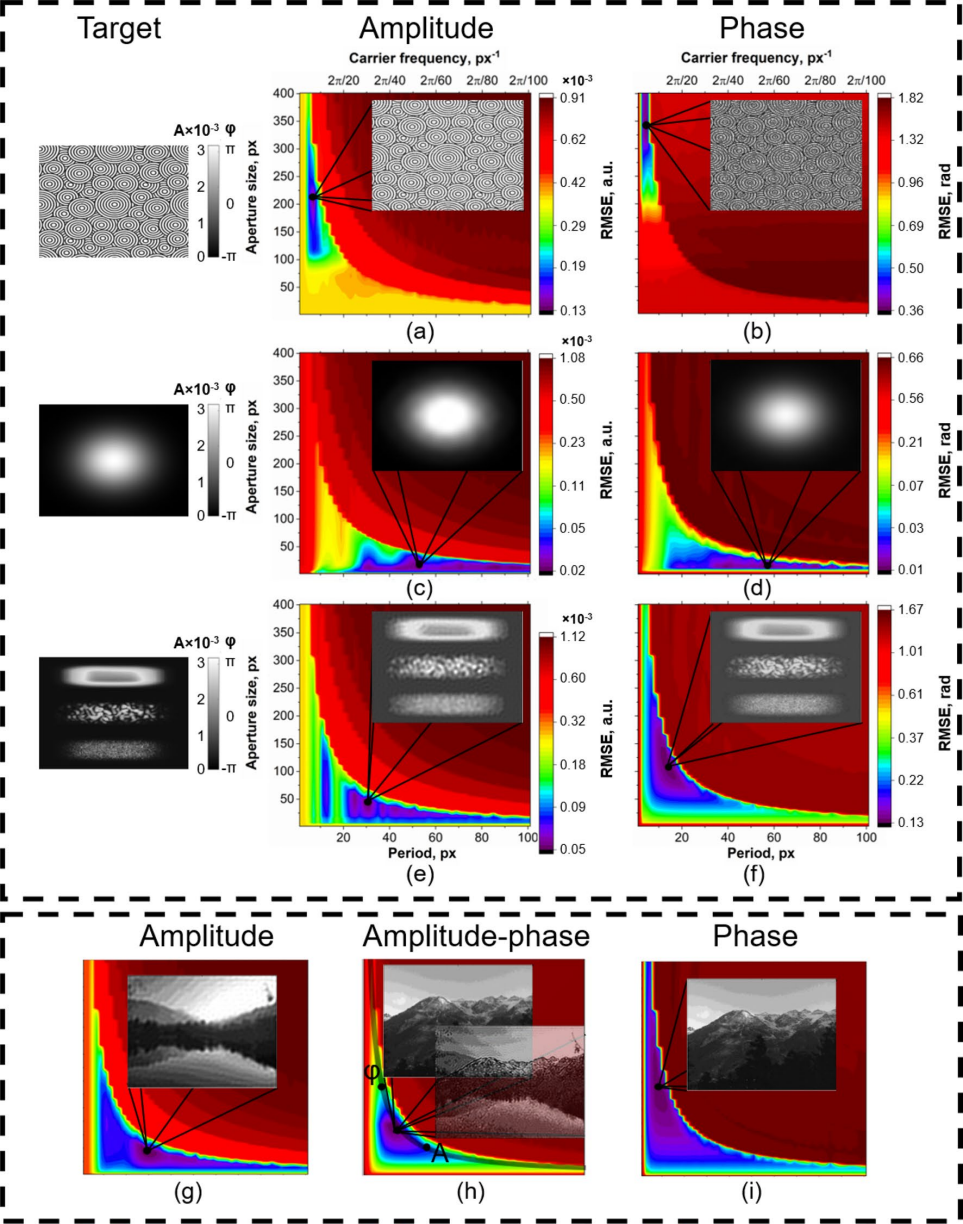
Wavefront modulation error maps for amplitude, phase and amplitude-phase modulation dependent on aperture size and fringe period for different objects. Insets: enlarged fragments, obtained intensity and phase distributions at the minimum RMSE points indicated by the markers. The upper frame shows amplitude (**a**,**c**,**e**) and phase (**b**,**d**,**f**) modulation error maps for objects “circles”, 2D Gaussian distribution and mixed-frequency phase object. The target distributions are shown on the left. The lower frame shows modulation error maps of amplitude (**g**), phase (**h**), and amplitude-phase (**i**). The minimal RMSE for amplitude-phase modulation lies approximately in the medium of amplitude and phase optimal parameters.

Let us consider the encoding of a few representative target distributions that contain a different range of spatial frequencies and intensity levels. The object “circles” is used as a demonstration of the small elements’ content influence on optimal aperture size and fringe period selection. The object 2D Gaussian distribution is used to demonstrate the example of high image quantization and low requirements to spatial resolution. A phase object characterized by high and low spatial frequencies content (mixed-frequency phase object)^53^ is an example of complex distribution with almost equal requirements to spatial resolution and quantization. Figure 4 shows the modulation error maps of amplitude and phase in the case of amplitude-only and phase-only field variations, respectively, for these objects. The insets show examples of the obtained intensity and phase distributions at the optimal parameters corresponding to the minimum error. Note that all the phase patterns suggest modulation within the range from $-\pi$:$\pi$ values. Strong variation of the phase value range may result in a significant change of the parameters for optimal complex wave modulation.

At first, we consider the extreme examples requiring either high spatial resolution or high quantization. For complex wave modulation with patterns of “circles” , it is important to achieve the maximum possible aperture size to provide sufficient spatial resolution. The target image includes repetitive circles of several fixed sizes. At the same time, for the 2D Gaussian example, a decrease in the distance between diffraction orders and the aperture size does not significantly deteriorate the image quality due to the absence of small details in this image. As shown in Fig. 4a,b, the minimal error area is local, and the global minimum corresponds to large aperture size and small fringe period. In the case of 2D Gaussian distribution, the high error of amplitude and phase modulation corresponds only to small fringe periods (high carrier frequencies) (Fig. 4c,d). The minimum error of wavefront modulation can be achieved in the case of small aperture size and large fringe period.

In the case of mixed-frequency object parameters, corresponding to optimal complex wave modulation, significantly depends on the type of modulation, amplitude or phase (Fig. 4e,f). In both cases, the minimum modulation error corresponds approximately to the average size of the aperture and period. However, for the amplitude-type modulation the minimum is shifted towards higher periods, and for phase modulation—towards higher aperture sizes. This can be explained by the presence of both high spatial frequencies, i.e., requirements for spatial resolution, and the presence of low spatial frequencies; the main part of the distribution is occupied by the band corresponding to the low-frequency elements with corresponding phase values $\pi$ and $-\pi$.

We considered the cases where only phase or only amplitude modulation took place. However, our DMD-based modulation system allows for simultaneous and independent amplitude-phase modulation, which provides unique opportunities for light manipulation in comparison with other techniques, e.g., based on liquid crystal-based spatial light modulators. Therefore, the case of simultaneous amplitude and phase modulation should be considered as well. Objects in both amplitude and phase were selected to investigate independent amplitude-phase modulation. The amplitude error, phase error, and modulation error together with the resulting intensity and phase distributions are shown in Fig. 4g–i.

Figure 4g shows that for the amplitude modulation of a complex object the area of minimum errors is shifted towards larger periods. The phase modulation optimum of the other complex distribution is located in the area of larger apertures (Fig. 4h). The modulation error minimum for independent amplitude-phase modulation is located in the middle of the graph (Fig. 4i), that is, approximately in the middle between the optimums for amplitude and phase modulation separately. Consequently, in the case of encoding complex waves that require both spatial resolution and quantization, it is necessary to choose the average values of the aperture size and fringe period.

In general, the area of minimal error for all distributions is below the hyperbola-type curve defined by the ratio between coefficient which depends on the image size, the optical system parameters, and the binary pattern period. Therefore, regardless of the type of distribution, the optimal ratio must be sought below this curve. Furthermore, optimal parameters for amplitude-phase modulation lie approximately in the middle of the optimal parameters of amplitude-only and phase-only modulation for the distributions. Hence, we will examine in more detail the dependence of the location of the optimum on the modulation type.

### Statistical study on modulation type and target size

The results presented above demonstrate a strong correlation between image quality and aperture size and carrier frequency or fringe period. Dependent on the target wavefront parameters, various binary patterns with different carrier frequencies should be generated. In this section, the influence of the modulation type will be considered in the context of a statistical study of different types of images.

**Figure 5:**
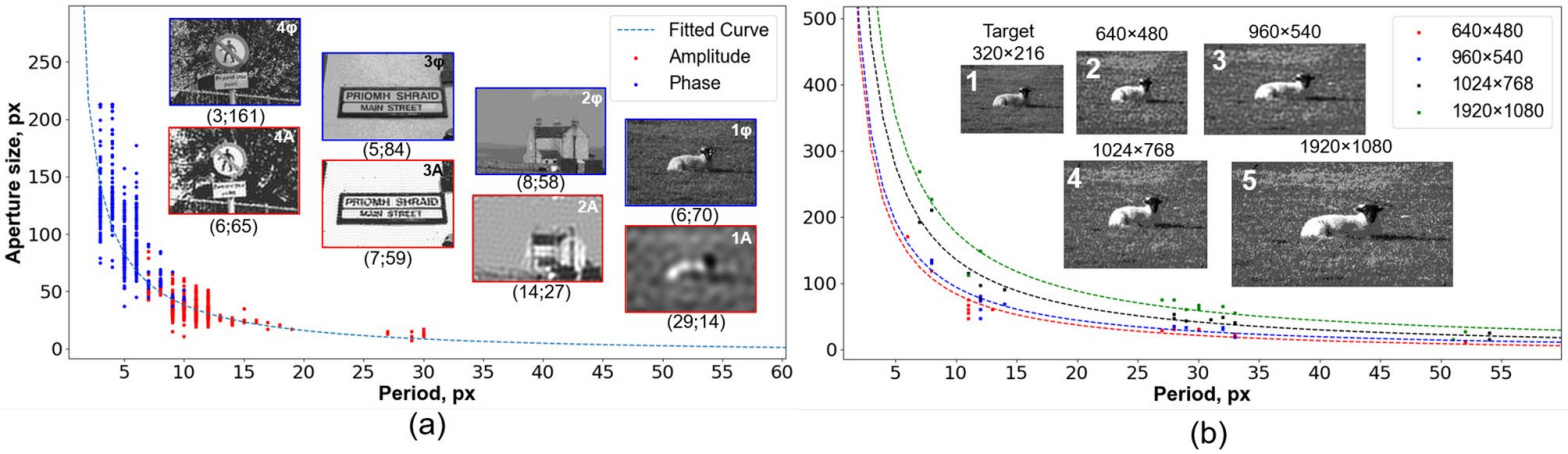
Minimal RMSE graph for amplitude-type and phase-type modulation. Approximation curves are plotted using specified points and are of hyperbola type. (**a**) The minimum RMSEs for dataset^54^ are indicated with red (amplitude-type) and blue (phase-type) dots. The insets show amplitude (red rectangles) and phase (blue rectangles) distributions, obtained using parameters which presented below each image in the form of (period; aperture size). (**b**) Minimal RMSE points for 10 different objects and different sizes equal to DMD matrix sizes for amplitude- and phase-type modulation. The insets show amplitude distributions, modulated using different DMD display resolutions which are presented above each image.

As was shown in Fig. 4, the minimum value of RMSE, namely, optimum, is located in some area of the error map, dependent on distribution type. To determine the area of minimum modulation error it is also necessary to consider the dependence of the optimum position on the type of modulation. We performed a numerical simulation of amplitude and phase modulation and found the minimum values of wavefront modulation error maps. A dataset consisting of 591 images^54^ was implemented. The distribution of the minimum error points is shown in Fig. 5a.

As can be seen from Fig. 5a, for amplitude-type modulation (red dots), the minimum error values lie in the area of large periods and small apertures, while the minimum error values for phase-type modulation (blue dots) are located in the area of the graph with large aperture size. This effect can be explained by the influence of the distribution of diffraction orders in the Fourier plane. In the case of amplitude-type modulation, only the intensity of the diffraction order varies, but not its form and direction. Phase-type modulation involves changing the form and direction of the first diffraction order, which is especially critical for significant shifts in the given phase distribution (e.g., from 0 to 2$\pi$). Thus, for encoding only the amplitude distribution, it is possible to increase the period of the binary pattern to achieve the required level of quantization, with small requirements for the size of the aperture. At the same time, the phase distribution encoding, especially containing high spatial frequencies and phase differences, requires increasing the aperture size, which entails the need to reduce the period to ensure, among other things, a sufficient level of quantization. As a consequence, the order of phase modulation errors is much higher than the order of amplitude modulation errors.

The few points corresponding to amplitude modulation with the binary fringe period of more than 25 pixels are optimums for images with frequent fluctuations in amplitude or phase (e.g., the grass in the inset image 1*A* in Fig. 5a). It is also seen that such a distribution is not resolved by amplitude modulation. This can be the result of the fact that such tiny details of the images a priori cannot be resolved within the given optical system. Meanwhile, the phase-type modulation of the same distribution allowed us to obtain sufficient spatial resolution. The insets also demonstrate the amplitude and phase distributions obtained at the indicated points. It can be seen that depending on the type of image (spatial resolution and quantization requirements), the optima are shifted along the hyperbola-type curve which represents the inverse dependence between quantization and spatial resolution. However, for distributions with small and frequent fluctuations, it is not possible to perform amplitude-type modulation due to the insufficiency of encoding pixels in a period. Therefore, we decided to analyze the impact of the target image size impact onto the global minima position on the wavefront modulation error maps. We performed a set of additional numerical experiments with different sizes of the same target amplitude and phase distributions, corresponding to various DMD array sizes available on the market.

Four DMDs with different display resolution were considered—DLP230GP (960 $\times$ 540), DLP7000 (1024 $\times$ 768), DLP6500 (1920 $\times$ 1080), and the first produced DMD^55^ (640 $\times$ 480), all of which were developed by Texas Instruments Incorporated. Wavefront modulation error maps were constructed for 10 images and optimal modulation parameters were found and plotted in Fig. 5b.

The dotted line shows the approximation curves that demonstrate the dependence of the position of the optimum relative to the same images modulated by different DMDs. Figure 5b shows that the approximation curve has a larger coefficient with increasing matrix size, that is, with increasing matrix size the aperture size and fringe period on the binary pattern increases. Depending on the type of image, the position of the optimum varies along the curve. In addition, the DMD cutoff frequency also affects image quality, especially for amplitude modulation. Using a small matrix (e.g., 640 $\times$ 480) for distribution with frequent amplitude variations (grass in Inset 1 in Fig. 5b, Inset 1A in Fig. 5a) imposes a constraint on the minimum fringe period, which must be large enough to encode all fluctuations. This results from the principle of amplitude modulation determined by the pixel occupancy rate. In other words, to encode frequent changes in amplitude in a binary pattern, frequent changes in the pixels that are On and Off are observed, which is impossible with insufficient display resolution. In addition, a much smaller aperture size is required in this case, which leads to a decrease in spatial resolution. However, using a DMD with larger matrices allows for encoding smaller amplitude fluctuations for the same distributions (insets 3–5 in Fig. 5b). At the same time, phase modulation is performed by changing the slope of the binary pattern fringes. Therefore, it is not necessary to utilize a large fringe period to encode small phase fluctuations. Thus, the DMD matrix size is directly related to the quality of the obtained amplitude images, however, the phase modulation is less dependent on the cutoff frequency (Inset 1A and 1$\varphi$ in Fig. 5a).

As the distance between the diffraction orders in the frequency space is proportional to the carrier frequency of the binary pattern as well as to the pixel size, the pixel size in numerical simulation is assumed to be 1, at carrier frequencies $k_x = k_y = k$, respectively, to calculate the hyperbola coefficient *a* it is necessary to count the product of the DMD matrix diagonal to the pixel size. Thus, this curve will be an envelope above which all parameters can be disregarded since the interference of adjusting diffraction orders will be observed in the detection plane.

### Experimental approbation of the optimization algorithm

In the sections presented above, the factors affecting the quality of amplitude or phase images were discussed. On this basis, optimization of the parameters of the binary pattern and the experimental setup was conducted for the experimental validation of numerical simulation.

**Figure 6:**
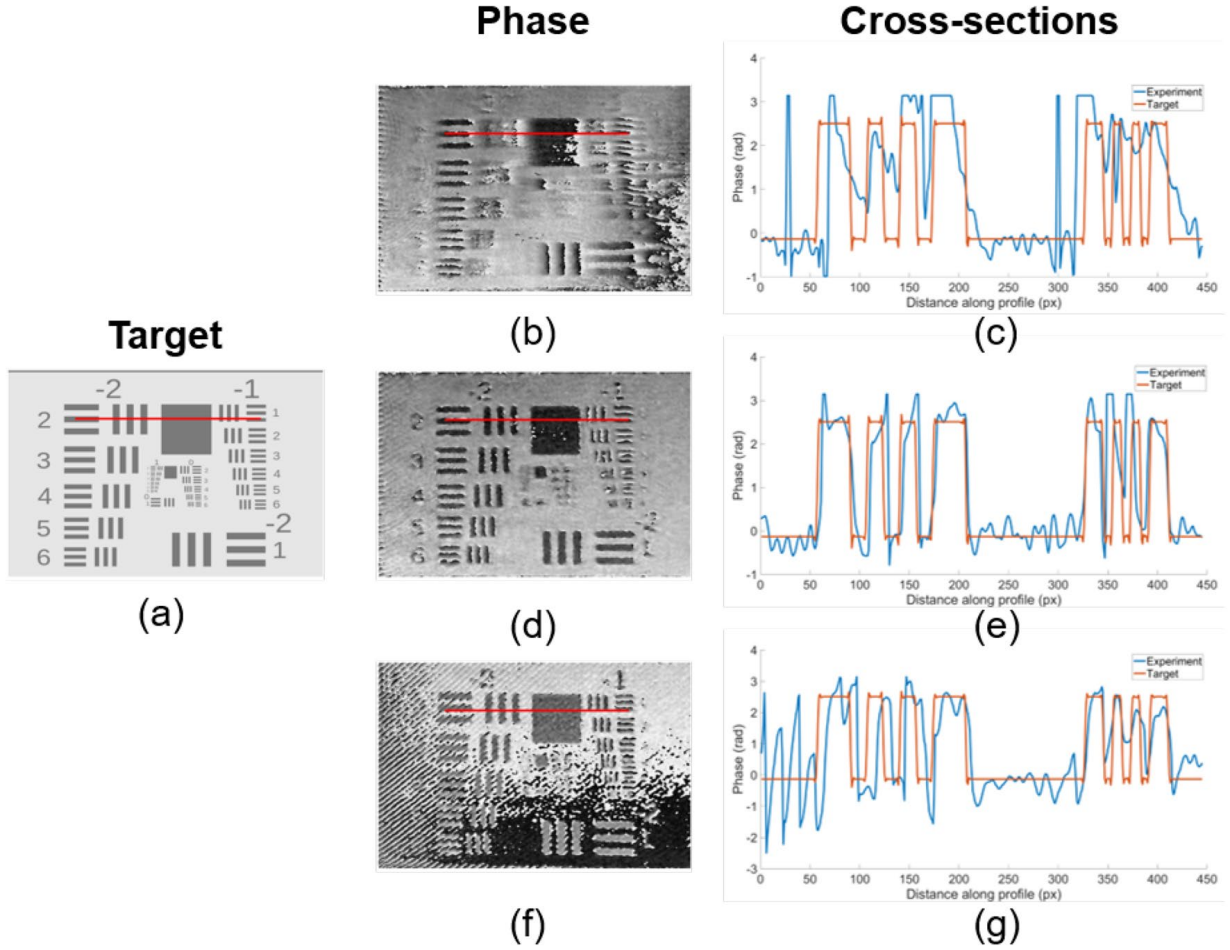
The results of phase distributions reconstruction with different filtering aperture sizes. (**a**) Target phase distribution; (**b**,**d**,**f**) reconstructed phase distributions with aperture size 1.5 mm, 2.25 mm and 3.0 mm, respectively, are shown in the first column. The red lines indicate the coordinates of cross-sections; the cross-sections (**c**,**e**,**g**) of the reconstructed phase distributions and target distribution are shown in the second column.

Experimental confirmation of the image reconstruction quality dependence on aperture size was performed on the example of phase-only modulation of the USAF 1951 test chart. This object is usually applied to determine the spatial resolution of optical systems and can be used successfully to demonstrate the impact of aperture size on the spatial resolution of the modulated wave. Since this object should be reconstructed with the highest possible spatial resolution, the setup parameters and the binary pattern were selected according to the optimization results. First, the distance between adjusting diffraction orders can be calculated as the ratio of the diagonal of the DMD matrix to the carrier frequency of the pattern. Then, as we perform phase-type modulation, the optimum will be located in the area of larger apertures and smaller periods. As many phase gradations are not required in this case, it is sufficient to proceed from the capabilities of the particular experimental setup. In our case, the minimum period of the binary pattern was 7 pixels. Therefore, the distance between diffraction orders is approximately 2.4 mm. We performed three experiments with different aperture sizes, presented in Fig. 6.

To confirm the accuracy of the selected parameters, the aperture size for filtration was changed in the *x* axis. The micrometer-adjusted monochromator slit was set in the Fourier plane to filter the first diffraction order. It allowed us to precisely vary the size of the filtration area along the *x* coordinate. The variation of only a single coordinate spatial aperture enables the demonstration of the impact of the aperture size.

The results of the phase distributions reconstruction at different filtering aperture sizes are shown in Fig. 6. Whereas the size was changed along the *x* axis, image blurring using the insufficient aperture size is observed only in the horizontal direction (Fig. 6a). Figure 6e shows the image deterioration due to adjacent diffraction orders passing through the filtration aperture. As can be seen in Fig. 6c, the optimal aperture size allowed us to reconstruct the phase distribution with minimal error and high image quality. It allowed us to detect the minimal resolvable pattern in the reconstructed phase distribution and estimate the spatial resolution. The spatial resolution was calculated using the following equation: $Resolution=2^{Group Number+\frac{Element Number-1}{6}}$. As can be seen from Fig. 6d, the minimal resolvable elements were “− 1” for Group Number and “3” for Element Number. The limiting resolution of an optical application setup was estimated as 0.63 lp/mm, which indicates a resolution of 793.7 μm. As the ratio between the real USAF test chart size and the DMD matrix size was 13.84, the length and width of the target lines in the optical system decreased proportionally, which resulted in a spatial resolution of approximately 57 μm.

The cross-sections comparing the target and reconstructed distributions are provided (Fig. 6c,e,g). In the case of the optimal aperture, an almost complete phase coincidence is observed (Fig. 6d). For a small aperture, there are no sharp edges between the objects, which is demonstrated in Fig. 6b. For large apertures, the image corrupting fringes resulting from the adjacent diffraction orders passage can also be seen in the cross-section in Fig. 6f).

The experimental results have demonstrated that the parameters of the binary pattern and aperture size, selected by the method described above, allowed us to reconstruct the phase with high image quality The correlation between aperture size and image quality was also confirmed.

## Discussion

Over the past few decades, wavefront manipulation by DMD has been widely used in light processing technology, despite the fact that DMD itself can be used for binary modulation of amplitude distribution only. However, the independent amplitude and phase modulation using DMD can be achieved with specific methods, such as computer-generated Lee holography^34^. Here, we proposed an approach for the optimization of the DMD-based amplitude and phase modulation based on the analysis of the target wave size, the requirements for spatial resolution and quantization, and modulation type.

The obtained results show that different types of target amplitude and phase distributions require different experimental parameters (i.e. binary fringe period and filtration aperture size) depending on the image type. Furthermore, all the analyzed target amplitude and phase distributions demonstrate the best modulation quality when the values of the mentioned experimental parameters lie below the hyperbola-type curve. In general, amplitude-type modulation of the incident wavefront typically requires higher fringe periods, whereas phase-type modulation requires larger filtration apertures. Moreover, larger target images for wavefront modulation allow us to obtain a better spatial resolution in terms of amplitude modulation.

To determine the optimal parameters for a particular case, it is necessary to consider several aspects of a target distribution. Initially, the size of the DMD matrix is taken into account. In the case where the binary pattern is generated with equal carrier frequencies in both directions, the limiting distance between the diffraction orders is calculated. Thus, we can determine the limiting coefficient of the hyperbola, above which all parameters will be suboptimal due to the superposition of diffraction orders. Further, it is necessary to proceed from the type of modulation. For amplitude modulation to find the optimal parameters we need to consider only the upper part of the hyperbola, and in the case of phase modulation, only the lower part. Finally, it is necessary to determine the ratio of the need to preserve spatial resolution and quantization. In the case of the predominance of the need to detail small elements of the distribution, the area of optimum will be shifted towards large apertures and small periods. In contrast, if the need to preserve quantization (e.g., when compensating for aberrations) prevails, the optimum is shifted toward larger periods. One critical point is the technical aspect. It is physically rather difficult to perform spatial filtering for patterns with small periods. Thus, the algorithm is as follows: (1) to determine the critical values of apertures and periods according to the formula, above this hyperbola values are not considered; (2) to consider type of modulation, for amplitude—above the hyperbola, for phase—below; (3) to determine the ratio of spatial resolution and image quantization requirements, depending on the task; (4) to take average values between certain parameters separately for the amplitude-type and the phase-type modulation in the case of the amplitude-phase modulation.

The correlations between quantization and carrier frequency, resolution and aperture size are particularly interesting, taking into account the wide variety of applications utilizing DMD. For instance, DMD patterns with lower spatial frequencies are used to correct aberrations by increasing the fringe period and decreasing the filtering aperture. Laser processing^14^, optoacoustic microscopy^19^ or optical coherence tomography^56^ require increased accuracy and high spatial resolution, which can be achieved by increasing the filtering aperture and decreasing the fringe period of binary hologram. In the case when the target wavefront poses a slow variation of amplitude and phase distributions, e.g., cells or intracellular organelles, quantization of modulated complex wave becomes important. Thus, in applications of such type, it is impossible to sacrifice one parameter to improve another, and it is necessary to maximize the spatial resolution and quantization of images equally.

## Conclusions

The results reported here demonstrate that the achievement of the accurate wavefront modulation using digital micromirror device requires optimization of experimental parameters according to the target complex wave, size of the distribution, its properties related with spatial resolution or quantization levels, and the type of modulation, i.e. amplitude, phase, or amplitude and phase. The mathematical and physical principles that enable independent manipulation of phase and amplitude were described and discussed in detail. Moreover, we have analyzed restrictions related to the distribution of the original information capacity of the encoding binary pattern and demonstrated that a trade-off between the spatial resolution of the encoded complex wave and its quantization should be achieved in dependence on the target complex wave. We have proposed an approach to opimize the generated DMD binary patterns for each amplitude and phase distribution. The approach is based on a simulation of the generation of modulated complex waves and takes into account the target wave size, the modulation type, and the requirements of the target distribution for quantization and spatial resolution.

The main applications of DMD for wavefront modulation, in which it is likely to be beneficial to maximize quantization or spatial resolution, are highlighted. Being optimized with the best experimental parameters, wavefront manipulation using DMD enables the accurate independent modulation of amplitude and phase distributions, and provides outstanding opportunities in various biomedical and technological applications. This technique is promising for implementation, for instance, in the fast generation of shaped beams^57^ or in studies of scattering media, for modulation of ultrashort laser irradiation^58,59^. However, the best results can be achieved only when the DMD pattern generation algorithm is optimized for certain types of amplitude and phase images, specific for a given application.

## Supplementary Information

Supplementary Information.
