# Supplementary material for: Optimization of DMD-based independent amplitude and phase modulation by analysis of target complex wavefront
Source: Sci Rep. 2022 May 11;12:7754. doi: 10.1038/s41598-022-11443-x (PMC9095630; doi:10.1038/s41598-022-11443-x)
Supplement: Supplementary file 1 — Supplementary Information. [file 41598_2022_11443_MOESM1_ESM.pdf]

Supplementary 1

Impact of experimental settings on the quality of the resulting wavefront

It was previously described how a binary pattern is generated for independent modulation of amplitude and phase distributions. However, for practical applications, the quality of the resulting wavefront, which is influenced by the experimental setup parameters, is crucial. One of the most important steps of modulation is the spatial filtration of one diffraction order. A concave lens is usually utilized for this purpose, in the focal plane of which the spatial filtering is performed.

In this section, the modulated wavefront quality is discussed and analyzed as a function of the 1st order filtration aperture size, and carrier frequency of binary hologram. To demonstrate the impact of experimental parameters on quality of the reconstructed wavefront, the “mountain” and “sky” images (Fig. 1) were taken as the target amplitude and phase distributions. These two images were chosen since the impact of both gray level quantization and spatial resolution can be visible on them

If the target amplitude and phase distributions have a large number of small details, the 1st order filtration can be a challenging problem due to the presence of high spatial frequencies overlapping with other diffraction orders. In this case, target wavefront blurring will be observed due to the inevitable loss of high spatial frequencies and corresponding data of small details. However, even under these conditions, it is possible to find the optimal aperture size, which enables obtaining a wavefront with minimal error. Fig. 1 demonstrates the impact of filtration aperture size and carrier frequency or fringe period variation on the generated complex wave and reveals the relationship between these parameters and image quality.

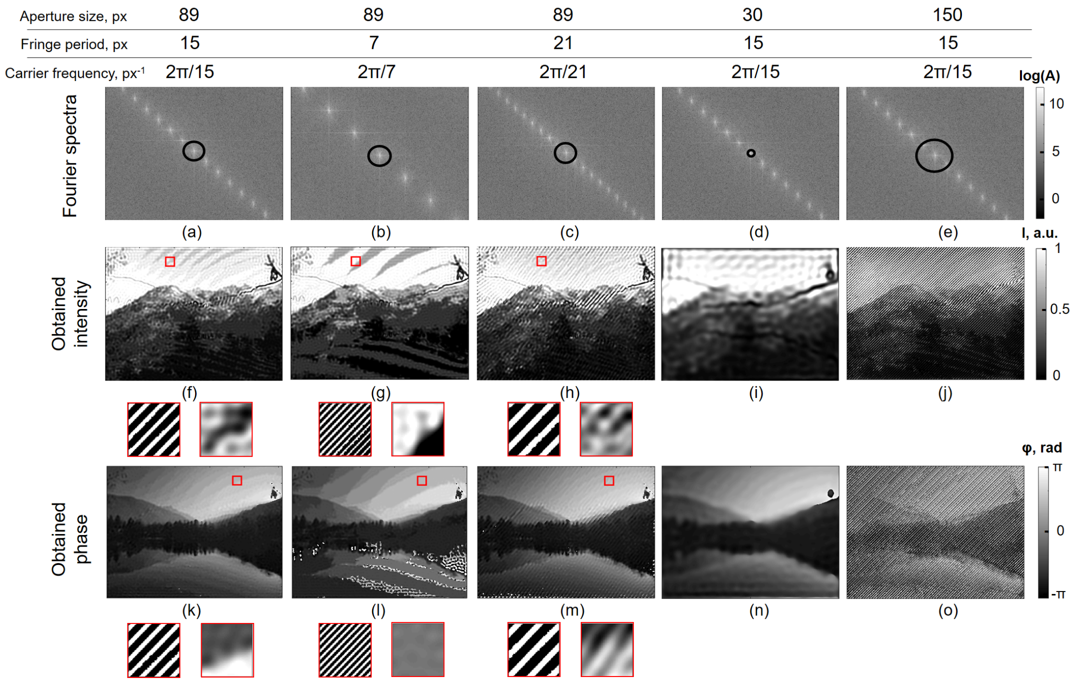

Figure 1. Dependence of reconstructed image quality on aperture size and fringe period (carrier frequency). Fourier spectra with indicated 1st order filtration apertures of different sizes (black circles) (a – e). The spectra are shifted so that the 1st diffraction order is located in the center; obtained intensity distributions (f – j). Obtained phase distributions (k – o). Insets: enlarged fragments of corresponding DMD patterns and fragments of phase or intensity distributions.

The first column of Fig. 1 represents the case with an optimal aperture size and fringe period. Very small filtration area (Fig. 1(d)) eliminates high spatial frequencies, which results in image blurring, as it can be seen in the fourth column (Fig. 1 (i, n)). On the other hand, excessively large aperture size (Fig. 1 (e)) leads to the adjacent diffraction orders penetration through the filtration aperture and the image quality reduction by defect appearance (Fig. 1 (j, o)).

To simplify the filtration and increase the aperture size, the distance between diffraction orders can be increased by the increment of the carrier frequency. The dependence between binary fringe periodicity and image quality was analyzed during carrier frequency variation. The second and third columns of Fig. 1 demonstrate filtration of the 1st diffraction order in Fourier plane and obtained amplitude and phase distributions at various values of carrier frequency. These results show a significant

improvement in spatial resolution due to the carrier frequency increase. However, the increase of carrier frequency is associated with the decrease of the average fringe period as well. The modulation of the wavefront amplitude and phase is possible due to the local variation of (1) the On pixel occupancy coefficient and (2) fringe period. The latter is related with the image quantization and its alteration results in the inevitable variation of the number of amplitude levels of the obtained distribution, as it can be seen in Fig. 1 (g, l, h, m). The explicit demonstration of the correlation between the fringe period and the image quantization is shown in insets for intensity distributions. Since the amplitude modulation is based on the variation of the On pixel occupancy coefficient, only six combinations of amplitude values can be encoded for fringe period of 7, while for the fringe period of 21 enables the encoding of 20 various values of the target amplitude (see insets for Fig. 1 (f, g, h)). Similarly, the phase value in the local area can be changed by variation of the fringe period. However, if the typical fringe period is small, it cannot be neither significantly decreased nor increased, which results in limited capability for phase value variation and low quantization of phase shift. Therefore, in the case of lower carrier frequency (larger fringe period), the image quantization level is high. This effect is also demonstrated in insets for the obtained phase distributions. For example, in insets for Fig. 1 (l) only one phase value is encoded (one color), while for insets for Fig. 1 (k), it is clear that there are at least two phase values. Furthermore, the fringe period in inset for Fig. 1 (m) changes by 1-2 pixels, allowing the smaller phase variations to be encoded. Therefore, increasing the fringe period (reducing carrier frequency) enables encoding smaller variations in the amplitude and phase of the wavefront, while complicating the first diffraction order filtering.

Thus, it was shown that the increase of carrier frequency results in higher spatial resolution but lower amplitude and phase quantization. Therefore, in the general case of DMD-based wavefront manipulation, the trade-off between two image quality compounds, namely, spatial resolution and quantization, should be sustained. If high spatial resolution is required for the modulated wavefront, a large enough carrier frequency of the binary pattern should be chosen to increase the distance between diffraction orders. However, if the target complex wave suggests smoothly varying amplitude and phase distributions, the carrier frequency should be limited to increase amplitude and phase quantization. Moreover, such a trade-off between these two image properties can be easily understood as the binary diffraction pattern may contain a limited amount of initial information about the target wavefront. More information can be provided either about spatial resolution (by the increase of carrier frequency) or quantization (by the decrease of carrier frequency), although both of these complex wave characteristics cannot be improved at the same time.
